# DOCLASP - Docking ligands to target proteins using spatial and electrostatic congruence extracted from a known holoenzyme and applying simple geometrical transformations

**DOI:** 10.12688/f1000research.5145.3

**Published:** 2016-06-16

**Authors:** Sandeep Chakraborty

**Affiliations:** 1 Plant Sciences Department, University of California, Davis, CA, 95616, USA; 2 Department of Biological Sciences, Tata Institute of Fundamental Research, Mumbai, 400 005, India; 3 Celia Engineers, Navi Mumbai, India

**Keywords:** protein, docking ligand, congruence

## Abstract

The ability to accurately and effectively predict the interaction between proteins and small drug-like compounds has long intrigued researchers for pedagogic, humanitarian and economic reasons. Protein docking methods (AutoDock, GOLD, DOCK, FlexX and Glide to name a few) rank a large number of possible conformations of protein-ligand complexes using fast algorithms. Previously, it has been shown that structural congruence leading to the same enzymatic function necessitates the congruence of electrostatic properties (CLASP). The current work presents a methodology for docking a ligand into a target protein, provided that there is at least one known holoenzyme with ligand bound - DOCLASP (Docking using CLASP). The contact points of the ligand in the holoenzyme defines a motif, which is used to query the target enzyme using CLASP. If there are significant matches, the holoenzyme and the target protein are superimposed based on congruent atoms. The same linear and rotational transformations are also applied to the ligand, thus creating a unified coordinate framework having the holoenzyme, the ligand and the target enzyme. In the current work, the dipeptidyl peptidase-IV inhibitor vildagliptin was docked to the PI-PLC structure complexed with myo-inositol using DOCLASP. Also, corroboration of the docking of phenylthiourea to the modelled structure of polyphenol oxidase (JrPPO1) from walnut is provided based on the subsequently solved structure of JrPPO1 (PDBid:5CE9). Analysis of the binding of the antitrypanosomial drug suramin to nine non-homologous proteins in the PDB database shows a diverse set of binding motifs, and multiple binding sites in the phospholipase A2-likeproteins from the Bothrops genus of pitvipers. The conformational changes in the suramin molecule on binding highlights the challenges in docking flexible ligands into an already ’plastic’ binding site. Thus, DOCLASP presents a method for ’soft docking’ ligands to proteins with low computational requirements.

## Introduction

The ability to computationally predict protein-ligand interactions with accuracy is an invaluable asset, since it allows for large scale screening at minimal costs ^1, 2^. Consequently, computational methods that predict the favorable conformation of a protein-ligand complex have been the focus of intense research over the last few decades ^3^. Protein docking methods are a subset of these methods, characterized by their ability to score a large number of possible conformations using fast algorithms. Among these, five programs - AutoDock ^4^, GOLD ^5^, DOCK ^6^, FlexX ^7^ and Glide ^8^ - are the most cited, although it should be noted that ‘the number of citations of a given paper is no measure of quality of the corresponding protein-ligand docking software program’ ^9^. Typically, a protein-ligand docking program has two distinct phases - conformational sampling (or searching) and scoring ^10^. Despite the significant progress in the field, there are several challenges arising from protein or ligand flexibility, entropic considerations or the presence of water molecules that need to be addressed ^11^.

Previously, the conservation of spatial and electrostatic properties in cognate pairs of residues in the catalytic site of proteins with the same functionality has been used to develop a computational method (CLASP) for detecting binding and catalytic sites ^12– 15^. In the current work, this methodology has been extended by proposing a method for docking ligands into target proteins - DOCLASP ( **Do**cking using **CLASP**). DOCLASP takes as input a set of proteins with known structures which bind a particular ligand, and a target protein into which the ligand is to be docked. Each of these holo structures is used to define a motif consisting of the first four residues making non-hydrophobic interactions. These motifs are used to query the target protein, using an enhanced version of the search engine used by CLASP that uses precompiled databases ^16^, and significant congruent matches are identified. These significant matches in the target protein are now superimposed to the binding residues (the motif) in the corresponding holoenzyme(s), thus creating a unified coordinate framework formed by the holoenzyme, the ligand and the target enzyme. This gives us the docked ligand to the target protein, which is outputted as a Pymol formatted file. In essence, the holoenzyme is replaced with the target enzyme if the contact points have a good spatial and electrostatic match in the target enzyme by aligning the congruent atoms. Thus, DOCLASP leverages the implicit search and scoring functions in CLASP to rank possible conformations.

The native activity of phosphoinositide-specific phospholipase C (PI-PLC) was previously shown to be inhibited by two dipeptidyl peptidase-IV (DPP4) inhibitors - vildagliptin (LAF-237) at micromolar concentrations, and K-579 at nanomolar concentrations using *in vitro* experiments based on CLASP analysis ^15^. Since ‘comparing docking programs can be difficult’ ^9^, the DOCLASP methodology is validated by docking vildagliptin to the PI-PLC structure in complex with myo-inositol ^17^. The docked ligand is free from steric clashes and interacts with the exact side chain residues that bind myo-inositol, providing corroboration of the validity of the proposed methodology. Next, an inhibitor of polyphenol oxidase (PPO) ^18^ was docked to the solved structure of a PPO from walnut ^19^, corroborating previous docking results from a modelled structure of the same protein ^20^. Finally, the promiscuous binding of suramin, a well-known antitrypanosomial drug ^21^, to nine non-homologous proteins in the PDB database revealed diverse binding motifs, and multiple binding sites even within phospholipase A2-like proteins from the Bothrops genus of pitvipers ^22^. Also, the conformational changes in suramin upon binding underscores the complexity of docking algorithms, which must sample a much larger conformational space created by both the changing binding site residues and ligand ^23^. Thus, the current work presents a fast methodology for docking ligands into protein structures based on spatial and electrostatic congruence of known binding sites to putative binding targets.

## Materials and methods

Data for “DOCLASP - Docking ligands to target proteins using spatial and electrostatic congruence extracted from a known holoenzyme and applying simple geometrical transformations” solvedJrPPO1Docked.pdb: phenylthiourea docked to the solved structure of JrPPO1 (Polyphenol oxidase from walnut, PDBid:5CE9).
modelledJrPPO1Docked.pdb: phenylthiourea docked to the modelled structure of JrPPO1 (Polyphenol oxidase from walnut) using SWISSMODEL based on the PDBid:1BUG, since the solved structure was not available at that time.
PLA2dockedsuramin.p1m: suramin docked to the phospholipase A2-like protein from PDBid:3BJW from *Echis carinatus* (saw scaled viper), showing that it has multiple sites of suramin binding. Click here for additional data file. Copyright: © 2016 Chakraborty S 2016 Data associated with the article are available under the terms of the Creative Commons Zero "No rights reserved" data waiver (CC0 1.0 Public domain dedication).

DOCLASP uses the basic hypothesis of CLASP - the non-triviality of the spatial and electrostatic congruence in cognate pairs seen across different structures of the same catalytic function, which is extended to the related concept of ligand binding ^12^. It takes as input a set of *Z* proteins with known structures ( Equation 1) which bind a particular ligand ( *Lig*), and a target protein into which *Lig* is to be docked ( *P _target_*). Each of these *Z* holo structures is used to define a motif consisting of *N* (=4) residues ( Equation 2), taking the first four closest non-hydrophobic interactions into account ( Algorithm 1).

$$\Phi_{proteins}^{Lig}=\left\{P_{1},P_{2}\ldots P_{Z}\right\}\;\;\;\;\;(1)$$

$$\Phi_{motifs}^{Pi}=\left\{R_{1},R_{2}\ldots R_{4}\right\}\;\;\;\;\;\;(2)$$

**Algorithm 1. GetMotif(): Choose n closest atoms, excluding hydrophobic interactions**

   
**Input**: *Protein* :

   
**Input**: *Ligand* :

   
**Input**: *n*: number of closest atoms to choose

   
**Output**: *ϕ _motif_* = { *atom*_1_ … *atom _n_*}

   
**begin**

         /* Output Motif */

        
*ϕ _motif_* = ∅ ;

        /* Accepted atom pairs - exclude hydrophobic interactions*/

       
*ϕ _AcceptedAtomPair_* = [O-N, N-O, O-H, H-O, O-O, N-N, N-H, H-N, S-H, H-S] ;

       
*ATOMS _Lig_* = atoms of all residues of *Ligand* ;

       /* Initial radius in Å */

       
*Radius* = 2.5 ;

       
**foreach** *atom _i_ in ATOMS _Lig_* **do**

             
*ϕ _atoms_* = ProteinAtomsWithinRadiusOfLigandAtom( *Protein,atom _i_, Radius*);

             
**foreach** *atom _j_ in ϕ _atoms_* **do**

                   
**if** *atom _i_-atom _j_ is in ϕ _AcceptedAtomPair_* **then**

                         InsertInMotifSet( *atom _j_, ϕ _motif_*);

                         
**if** *(ϕ _motif_* == *n)* **then**

                               last ;

                         
**end**

                   
**end**

             
**end**

             /* increment radius by 0.1 Å */

             
*Radius* = *Radius* + 0.1;

       
**end**

       return *ϕ _motif_* ;

   
**end**

Each position of the motif has a set of amino acids specified to allow for stereochemically equivalent matches at that particular position ( Equation 3), such that while matching amino acid type of *r _i_* should belong to *GROUP _i_*.

$$\Phi_{groups}=\left\{GROUP_{1},GROUP_{2}\ldots GROUP_{4}\right\}\;\;\;\;\;(3)$$

Previously, the *K* sets of *N* residues were obtained in *P _target_* using an exhaustive search procedure similar to the one used in SPASM ^24^. An enhanced algorithm now precompiles all possible motifs of a set ( *N*=4 in this case) of predefined amino acid residues from a protein structure that occur within a specified distance ^16^, and selects the appropriate ones based on each motif ( Equation 4). Any match below a user defined threshold score ( *S _thresh_*) is discarded.

$$\begin{array}{l} \;\;\Phi_{matches}^{Pi}=\left\{M_{1}^{Pi},M_{2}^{Pi}\ldots M_{K}^{Pi}\right\},\\ \;\;\;\;\;\;\;\;\;\forall (j=1\ldots K)[M_{j}^{Pi}=\left\{r_{1},r_{2}\ldots r_{N}\right\},\forall (p=1\ldots N)[AminoAcidType(r_{p})\in GROUP_{P}]],\\ \;\;\;\;\;\;\;\;\;[CScore_{M_{1}^{Pi}}<CScore_{M_{2}^{Pi}},CScore_{M_{2}^{Pi}}<CScore_{M_{3}^{Pi}}\ldots ],\;\;\;\;\;\;\;\;\;\;\;\;\;\;\;\;\;\;\;\;\;\;\;\;\;\;\;\;\;\;\;\;\;\;\;\;\;\;\;\;\;\;\;\;\;\;\;\;\;\;\;\;\;\;\;\;\;\;\;\;\;\;\;\;\;\;\;\;\;\;\;\;\;\;\;(4)\\ \;\;\;\;\;\;\;\;\;CScore_{M_{K}^{Pi}}<S_{thresh}\;\;\;\;\;\;\;\;\;\;\;\;\;\;\;\;\;\;\;\;\;\;\;\;(4) \end{array}$$

In Case $\Phi_{matches}^{Pi}$ is null, the ligand *Lig* can not be docked to the target protein *P _target_*. $M_{1}^{P_{i}}$, the first element, has the minimum *CScore* and represents the putative binding site in *P _target_* based on the holoenzyme *P _i_*. The set of putative binding sites Φ _*bindsite*_ is thus defined ( Equation 5).

$$\Phi_{bindsite}=\{M_{1}^{P_{1}},M_{1}^{P_{2}}\ldots M_{1}^{P_{Z}}\}\;\;\;\;\;\;\;(5)$$

In order to compute CScore, the 3D distances and PD are computed and values are then compared with the corresponding feature values obtained from the holoenzyme motif. The distance scores are normalized since that the same pairwise distance deviation should count more when the reference distance is less. While scoring electrostatic potential differences, deviations < 100 are ignored. Similarly for higher potential differences, the deviations are more loosely constrained than for lower potential differences. The differences are absolute values.

Each element of Φ _*bindsite*_ ( $M_{1}^{P_{i}}$) is now superimposed to the corresponding holoenzyme, based on the motif binding *Lig* in *P _i_*. In order to superimpose these motifs, linear and rotational transformations are applied on all atoms such that the first three atoms lie on the same plane (Z=0), the first atoms are the origin of the coordinate axis and the second atoms lie on the Y axis. This creates a unified coordinate framework having the holoenzyme, the ligand and the target enzyme, thus providing the docked ligand in the target enzyme. Essentially, the holoenzyme is replaced with the target enzyme if the contact points have a good spatial and electrostatic match in the target enzyme by aligning the congruent atoms. This docked ligand is now outputted as a Pymol formatted file.

The DOCLASP package is written in Perl on Ubuntu. Hardware requirements are modest - all results here are from a simple workstation (2GB ram) and runtimes were a few minutes at the most. Adaptive Poisson-Boltzmann Solver (APBS) and PDB2PQR packages were used to calculate the potential difference between the reactive atoms of the corresponding proteins ^25, 26^. The APBS parameters and electrostatic potential units were set as described previously in 12. All protein structures were rendered by PyMol ( http://www.pymol.org/).

Previous CLASP analysis of the spatial and electrostatic properties of active site residues in PI-PLC from *B. cereus* indicated that it is a prolyl peptidase, which was also validated by *in vitro* experiments ^13^. Subsequently, it was shown that PI-PLC is inhibited by two dipeptidyl peptidase-IV (DPP4) inhibitors - vildagliptin (LAF-237) at micromolar concentrations, and K-579 at nanomolar concentrations. Since there are no DPP4 structures solved which ligand K-579, a DPP4 protein structure in complex with vildagliptin (PDBid:3W2TA) ^28^ provided the five closest atoms in the protein (E205, E206, S630, Y662 and Y547) (see Methods) that make non-hydrophobic interactions with the ligand ( Table 1).

**Table 1.** Residues in dipeptidyl peptidase-IV (DPP4) holoenzyme (PDBid:3W2TA) that have non-hydrophobic interactions with the bound DPP4 inhibitor vildagliptin. Interactions are sorted based on the distance. R/A/LA/D: Residue number/Atom of the residue/Atom of ligand/distance between the interacting atoms (in Å). For example, ‘S630/OG/N2/2.4’ means that the atom OG from Ser630 is at 2.4 Å from the N2 atom of vildagliptin in PDBid:3W2TA.

R/A/LA/D	R/A/LA/D	R/A/LA/D	R/A/LA/D	R/A/LA/D
S630/OG/N2/2.4	E205/OE1/N12/2.8	Y662/OH/O20/3	E206/OE2/N12/3	Y547/OH/N2/3.1

A subset of these atoms might be sufficient to ligand vildagliptin in the target protein. Thus, 54=5 motifs, each with four atoms, were created using the five closest atoms in the protein. The binding of ligands is known to induce electrostatic and spatial perturbations in the binding site. The spatial and electrostatic perturbations induced by the vildagliptin binding is shown by comparing the apo (PDBid:2OQIA) and the holoenzyme (PDBid:3W2TA) in Table 2. Hence, the electrostatic and spatial profile of the motif were obtained from the apo DPP4 enzyme (PDBid:2OQIA), and then used for querying the PI-PLC apo structure (PDBid:1PTDA).

**Table 2.** Changes in the conformation of the binding site due to vildagliptin (a DPP4 inhibitor) binding. We compare the pairwise distance and electrostatic potential difference (EPD) changes in the apo (PDBid:2OQIA) and holo (PDBid:3W2TA) enzymes. Note that the pairwise distance between these atoms change in the ligand free PDB (2OQIA) as compared to the protein with bound inhibitor (2BUBA). For example, the distance between E205OE2 and E206OE2 (pair ab) changes from 3.9 Å to 5.6 Å. Also, there is a definite change in the EPD between E205OE2 and E205OE2 (pair ab). D = Pairwise distance in Å. PD = Pairwise potential difference. The electrostatic potential are in dimensionless units of kT/e where k is Boltzmann’s constant, T is the temperature in K and e is the charge of an electron.

PDB	Active site atoms (a,b,c,d)		ab	ac	ad	bc	bd	cd
2OQIA apo 3W2TA holo	GLU205OE2,GLU206OE2,SER630OG,TYR662OH, GLU205OE2,GLU206OE2,SER630OG,TYR662OH,	D PD D PD	3.9 75.7 5.6 -74.4	7.9 -193.4 9.1 -307.9	4.0 -94.5 6.1 -159.8	10.1 -269.1 9.4 -233.5	3.5 -170.2 5.1 -85.5	7.3 98.9 5.6 148.1

These motifs were used to query the PI-PLC structure using an enhanced algorithm (PREMONITION) that precompiles all motifs in a database ^16^. Table 3 shows the best matches obtained in the PI-PLC structure for the five partial motifs. All these matches have significant electrostatic congruence. The root mean square deviation (RMSD) have low values - however, this is a deceptive metric since these deviations are averaged out. The maximum pairwise distance is another metric to discriminate the spatial congruence, and should be used in combination with the RMSD value. All of these above mentioned matches have significant maximum pairwise distance deviation. Also, three matches do not comprise of active site residues (motifs 2, 3 and 4). However, it can be seen that the first and fifth matches comprises of active site residues (involved in the binding of myo-inositol in PDBid:1PTGA), and have three residues (Asp67, Asp198 and Trp178) in common.

**Table 3.** Querying PI-PLC using partial motifs derived from the atoms in vildagliptin that make contact to the DPP4 enzyme (PDBid:3W2TA). The comparison is done using apo enzymes (PDBid:1PTDA for PI-PLC and PDBid:2OQIA for DPP4), since the binding of a ligand induces spatial and electrostatic changes in the active site. The fifth motif has the least rmsd deviation, and comprises of active site residues (involved in the binding of myo-inositol in PDBid:1PTGA). Out of the best matches in the other four motifs, three do not comprise of active site residues (motifs 2, 3 and 4). Motif 1 has a reasonably significant match, and has three residues (Asp67, Asp198 and Trp178) in common with the best match for Motif 5. These three residues from PI-PLC (Asp67, Asp198 and Trp178) and corresponding three residues from DPP4 (Glu205, Glu206 and Tyr662) were used to superimpose DPP4 and PI-PLC. N = Motif number. D = Pairwise distance in Å. PD = Pairwise potential difference. Rmsd = Root mean square deviation. Max = maximum pairwise distance deviation. APBS writes out the electrostatic potential in dimensionless units of kT/e where k is Boltzmann’s constant, T is the temperature in K and e is the charge of an electron.

PDB	N	Active site atoms(a,b,c,d)		ab	ac	ad	bc	bd	cd	Rmsd/Max (Å)
2OQIA 1PTDA	1	S630OG,E205OE1,Y662CZ,E206OE1 S113OG,D67OD1,W178CZ2,D198OD1	D PD D PD	9.5 237 4.3 195	7.8 -104 6.7 -79	11.2 228 10.1 277	7.2 -341 6.7 -275	6.6 -9 7.7 81	5.8 332 4.9 357	0.9/5.1
2OQIA 1PTDA	2	S630OG,E205OE1,Y662CZ,Y547CZ S244OG,D274OD1,Y275CZ,Y246CZ	D PD D PD	9.5 237 11.3 312	7.8 -104 7.2 -104	5.4 -241 8.1 -171	7.2 341 7.0 -417	11.3 -478 11.6 -484	9.3 -137 5.9 -67	0.8/3.3
2OQIA 1PTDA	3	S630OG,E205OE1,E206OE1,Y547CZ S129OG,D126OD1,E125OE1,Y164CZ	D PD D PD	9.5 237 7.3 172	11.2 228 12.5 262	5.4 -241 6.7 -232	6.6 -9 5.5 90	11.3 -478 12.9 -405	10.4 -469 17.4 -495	1.3/7.0
2OQIA 1PTDA	4	S630OG,Y662CZ,E206OE1,Y547CZ S244OG,Y275CZ,D274OD1,Y246CZ	D PD D PD	7.8 -104 7.2 -104	11.2 228 11.3 312	5.4 -241 8.1 -171	5.8 332 7.0 417	9.3 -137 5.9 -67	10.4 -469 11.6 -484	0.8/3.3
2OQIA 1PTDA	5	E205OE1,Y662CZ,E206OE1,Y547CZ D67OD1,W178CZ2,D198OD1,Y200CZ	D PD D PD	7.2 -341 6.7 -275	6.6 -9 7.7 81	11.3 -478 11.6 -318	5.8 332 4.9 357	9.3 -137 9.6 -42	10.4 -469 6.3 -400	0.7/4.0

**Figure 1.**
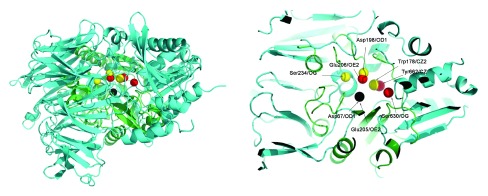
Superimposing PI-PLC (PDBid:3W2TA in green) and DPP4 (PDBid:2OQOA in cyan). Three residues from PI-PLC (Asp67, Asp198 and Trp178 in yellow) were superimposed to the corresponding three residues from DPP4 (Glu205, Glu206 and Tyr662 in red). Asp67OD1 and Glu205OE2 is at the center of the coordinate axis (coordinates = [0,0,0]) (in black), Asp198OD1 and Glu206OE2 lies on the X-Y axis (i.e. Y coordinate is 0) and Tyr662CZ and Trp178CZ2 are on the X-Y plane (i.e. Z coordinate is 0). Asp67, Asp198 and Trp178 in the PI-PLC protein overlaps well with Glu205, Glu206 and Tyr662 from DPP4, but the Ser234-Ser630 pair is not spatially congruent.

Thus, these three residues from PI-PLC (Asp67, Asp198 and Trp178) and corresponding three residues from DPP4 (Glu205, Glu206 and Tyr662) were used to superimpose DPP4 and PI-PLC. Table 4 shows the congruence of these residues. The corresponding holo structures - PDBid:3W2TA for DPP4, and PDBid:1PTGA for PIPLC - were used for the superimposition. This superimposition applies geometric transformations such that Asp67OD1 and Glu205OE2 were at the center of the coordinate axis (coordinates = [0,0,0]), Asp198OD1 and Glu206OE2 lies on the X-Y axis (i.e. Y coordinate is 0) and Tyr662CZ and Trp178CZ2 were on the X-Y plane (i.e. Z coordinate is 0). Figure 1 shows the superimposed proteins. It is observed that (Asp67, Asp198 and Trp178) overlaps well with (Glu205, Glu206 and Tyr662).

**Table 4.** Spatial and electrostatic congruence of a three residue partial motif. The match is significant and comprises active site residues (involved in the binding of myo-inositol in PDBid:1PTGA). Pair ‘ab’ is considered to be electrostatically congruent since the PD values are close to zero, and can be considered almost equipotential. This is expected for atoms of the same type from the same residue (GLU205OE1/GLU206OE1 and ASP67OD1/ASP198OD1). These three residues from PI-PLC (Asp67, Asp198 and Trp178) and corresponding three residues from DPP4 (Glu205, Glu206 and Tyr662) were used to superimpose DPP4 and PI-PLC. D = Pairwise distance in Å. PD = Pairwise potential difference. APBS writes out the electrostatic potential in dimensionless units of kT/e where k is Boltzmann’s constant, T is the temperature in K and e is the charge of an electron.

PDB	Atoms(a,b,c)		ab	ac	bc
2OQIA 1PTDA	GLU205OE1,GLU206OE1,TYR662CZ, ASP67OD1,ASP198OD1,TRP178CZ2,	D PD D PD	6.6 -9.3 7.7 81.8	7.2 -341.5 6.7 -275.7	5.8 -332.2 4.9 -357.6

**Figure 2.**
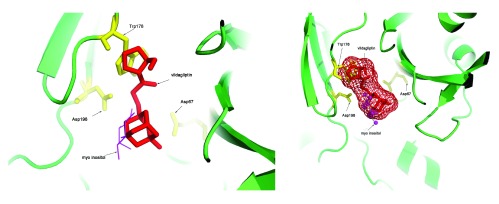
Docking vildagliptin to the PI-PLC structure in complex with myo-inositol (PDBid:1PTGA). It can be seen that vildagliptin fits into the binding site of PI-PLC. It also makes non-hydrophobic contacts to the residues in the protein similar to those made by myo-inositol ( Table 5).

These transformations were also applied to the vildagliptin molecule, and this resulted in a docked structure for this molecule into the PI-PLC protein. Figure 2 shows the vildagliptin docked into the PI-PLC structure which is complexed with myo-inositol (PDBid:1PTGA). The distances of the atoms in vildagliptin and myo-inositol that interact (excluding hydrophobic interactions) to the first ten residues in the PI-PLC structure are shown in Table 5. It is interesting to note that the residues shown in Table 5 are all part of side chain residues in close contact with the myo-inositol ring (shown in Figure 7 ^13^). Further validation was obtained by observing that both Arg69/NH1 and His32/NE2 interact with atom O4 in vildagliptin, and Arg69/NH2 and His32/NE2 interact with O2 in myo-inositol in the PI-PLC structure. The Pymol script for visualizing the docking (SupplementaryPymol.p1m) and a movie (SupplementaryMovie.avi) are also provided as Supplementary information.

**Table 5.** Atoms of myo-inositol and vildagliptin that make contact to the residues of the PI-PLC structure (PDBid:1PTGA). These interactions exclude hydrophobic interactions, and the first closest ten atoms are chosen. Out of ten, seven residues obtained by docking vildagliptin using DOCLASP are seen to be equivalent to those that are known to bind myo-inositol to the PI-PLC structure ^13^, while two more have the same amino acid type (marked by asterisks). Only one pair has a different amino acid type (Tyr200 for myo-inositol and Glu117 for vildagliptin). Further validation is obtained by observing that both Arg69/NH1 and His32/NE2 both interact with atom O4 in vildagliptin, and Arg69/NH2 and His32/NE2 interact with O2 in myo-inositol in the PI-PLC structure.

myo-inositol	vildagliptin	Same?
ASP198/OD1/O3/2.6 ARG163/NH2/O5/2.7 HIS32/NE2/O2/2.9 ARG69/NH2/O2/3.3 LYS115/NZ/O5/3.4 TRP178/NE1/O4/4.0 ASP33/OD1/O2/4.1 SER234/OG/O3/4.8 ASP180/OD1/O6/4.5 TYR200/OH/O6/4.4	ASP198/OD2/N12/3.1 ARG163/NE/O20/1.1 HIS32/NE2/O4/4.5 ARG69/NH1/O4/1.0 LYS115/NZ/O4/4.9 TRP178/NE1/N15/2.2 ASP33/OD1/O4/2.8 SER113/OG/O20/5.3 ASP67/OD2/O4/3.8 GLU117/OE1/O4/4.1	Y Y Y Y Y Y Y * * N

It is important to comment on the previous hypothesis of a nucleophilic serine being responsible for the inhibition of PI-PLC using vildagliptin. The electrostatic and spatial profile of the motif 4 from DPP4 is compared to the electrostatic and spatial profile of matching active site residues in PI-PLC in Table 6, including serine in the comparison. It can be seen that Ser630 in DPP4 has a significant spatial difference as compared to Ser234 in PI-PLC (pair ‘bc’ has a difference of 5 Å), and also a reasonable electrostatic difference (pair ‘ac’ has a difference of 144 PD units). The relatively large distance over which Ser234 in PI-PLC interacts with myo-inositol (4.8 Å) indicates that Ser234 is not directly involved in the binding of the ligand. However, it is responsible for creating the electrostatic milieu that is required for other interacting residues to attain their appropriate potential. Even for DPP4, many inhibitors do not interact with the nucleophilic Ser630 (manuscript in preparation) - although the vildagliptin molecule does ^28^. Thus, the previous conjecture of a nucleophilic serine being *directly* responsible for the binding of DPP4 inhibitors to PI-PLC, as implied by the catalytic triad congruence, is incorrect ^13^. However, this serine is indirectly responsible for driving the neighboring residues to an appropriate state. Spatial constraints are an additional discriminator.

**Table 6.** Potential and spatial congruence of the residues binding vildagliptin in DPP4 structure (PDBid:2OQIA) to the putative binding site in PI-PLC structure (PDBid:1PTDA). Both the structures are apo enzymes, since the binding of a ligand induces spatial and electrostatic changes in the active site. Ser630 in DPP4 has a significant spatial difference as compared to Ser234 in PI-PLC (pair ‘bc’ has a difference of 5 Å), and also a reasonable electrostatic difference (pair ‘ac’ has a difference of 144 PD units). D = Pairwise distance in Å. PD = Pairwise potential difference. APBS writes out the electrostatic potential in dimensionless units of kT/e where k is Boltzmann’s constant, T is the temperature in K and e is the charge of an electron.

PDB	Active site atoms(a,b,c,d)		ab	ac	ad	bc	bd	cd
2OQIA	GLU205OE1,GLU206OE2,SER630OG,TYR662CZ,	D PD	5.2 31.7	9.5 -237.4	7.2 -341.5	10.1 -269.1	4.2 -373.2	7.8 -104.1
1PTDA	ASP67OD1,ASP198OD1,SER234OG,TRP178CZ2,	D PD	7.7 81.8	8.2 -93.7	6.7 -275.7	5.7 -175.6	4.9 -357.6	9.2 -182.0

DOCLASP was also used recently ^29^ to dock human karyopherin to the VP24 ^30^ protein of the Reston Ebola strain using the VP24 from Zaire Ebola ^31^ as a template, and demonstrate that a single mutation might be one of the critical factors responsible for the non-pathogenic nature of Reston Ebola in humans ^32, 33^.

## Docking phenylthiourea to polyphenol oxidase from walnut

**Figure 3.**
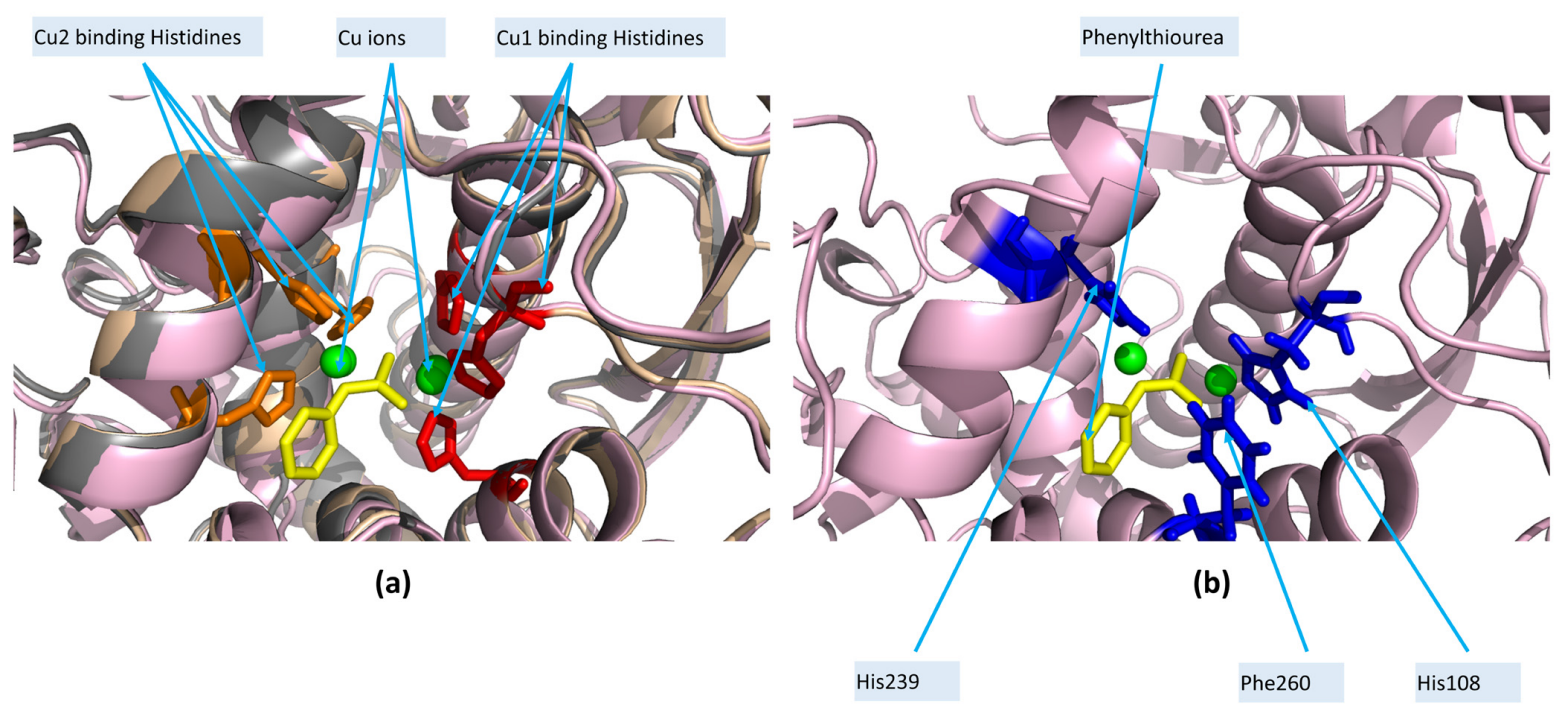
Docking phenylthiourea (URS) to polyphenol oxidase (PPO) from walnut based on the PPO from sweet potato (PDBid:1BUG). The copper binding histidines (His88/109/118 for Cu1) are in red and (His240/244/274 for Cu2) are in orange. URS in yellow, copper ions in green. ( **a**) Superimposition of PDBid:1BUG (wheat), modelJrPPO1 (grey) and solvedJrPPO1 (pink). ( **b**) Three closest residues in solvedJrPPO1 that ligand URS (His108/His239/Phe260) are in blue.

Polyphenol oxidases (PPO/tyrosinases/catechol oxidases) are copper enzymes implicated in the biosynthesis of quinones ^34^. The recently sequenced walnut genome sequence revealed the presence of two PPO genes (JrPPO1/2) ^20^, only one of which was previously known (JrPPO1) ^18^. Since there were no known structures of JrPPO1/2 at the time of writing ^20^, SWISSMODEL was used to model JrPPO1 (modelJrPPO1) based on the structure of the homologous PPO from *Ipomoea batatas* (PDBid:1BUG, sweet potato). The structure of JrPPO1 was recently solved (solvedJrPPO1, PDBid:5CE9) ^19^. DOCLASP docked phenylthiourea (URS) to modelledJrPPO1 and solvedJrPPO1 by aligning conserved copper binding histidines (His87, His108 and His117 in JrPPO1, Figure 3) (see modelledJrPPO1Docked.pdb and solvedJrPPO1Docked.pdb in Dataset1). The binding pose of URS showed a similar configuration in both modelledJrPPO1 and solvedJrPPO1 ( Table 7).

**Table 7.** Docking phenylthiourea (URS) to modelled (modelJrPPO1) and solved (solved- JrPPO1,PDBid:5CE9A) polyphenol oxidase (PPO) from walnut: JrPPO1 has been modelled using the PPO from *Ipomoea batatas* (sweet potato), where URS is liganded by His109/His240/Phe261/Asn260. After DOCLASP docking, modelledJrPPO1 has the analogous His108/His239/Asn259 as binding residues, while solvedJrPPO1 has His108/His239/Phe260 as binding residues.

PDB	PDBatom	URSatom	Distance°A
1BUGA 1BUGA 1BUGA 1BUGA 1BUGA	HIS/109/NE2 HIS/109/CD2 PHE/261/N HIS/240/CE1 ASN/260/C	N2 N2 C5 N1 C5	2.7 2.9 3.1 3.1 3.2
modelJrPPO1 modelJrPPO1 modelJrPPO1 modelJrPPO1 modelJrPPO1	HIS/108/NE2 HIS/108/CD2 HIS/239/CE1 HIS/108/NE2 ASN/259/C	N2 N2 N1 S1 C5	2.7 2.9 3.0 3.1 3.2
solvedJrPPO1 solvedJrPPO1 solvedJrPPO1 solvedJrPPO1 solvedJrPPO1	PHE/260/CE1 HIS/108/NE2 PHE/260/CD1 HIS/239/CE1 HIS/108/CD2	N2 N2 N2 N1 N2	2.4 2.4 2.6 2.8 2.9

## Analysis of suramin binding to a set of nine non-homologous proteins

Human African Trypanosomiasis (HAT), endemic to sub-Saharan Africa, is caused by the parasite Trypanosoma brucei and is transmitted via the tsetse fly ^35^. Suramin, a hexasulfonated naphthylurea, is a antitrypanosomial drug which has been in clinical use for decades, and more recently for the treatment of malignant tumors ^36– 39^. It has been known for a while now that suramin binds to several human proteins including cullin-RING E3 ubiquitin ligases ^40^, serum albumin ^41^, P2X receptors ^42^, neutrophil elastases ^43^, topoisomerase I and II ^44^, thrombin ^45^, receptor/G protein ^46^ and human secreted group IIA phospholipase A2 ^47^. Such interactions may be the rationale for its toxicity in several cases ^39^. Of late, there have been a plethora of instances highlighting the potential of suramin based therapeutics: treating severe fever with thrombocytopenia syndrome caused by bunyavirus (by inhibiting viral nucleocapsid protein) ^48^, malaria (by inhibiting falcipain-2, a cysteine protease) ^49^, gastroenteritis (by inhibiting RNA dependent RNA polymerase in Noroviruses) ^50^, EV71 infection ^51^, tuberculosis (by inhibiting RecA) ^52^ and Dengue virus infection (by inhibiting envelope protein binding to target cell heparan sulfate).

The current PDB database has nine non-homologous protein structures which ligand suramin ( Supplementary Table 1). Positively charged residues (Lys and Arg) in some proteins (PDBids:1Y4LB, 1Y8EA, etc) and negatively charged residues (Asp and Glu) in other proteins (PDBids:2H9TH, 3GANA, etc) interact with suramin ( Table 8). However, all possible hydrogen bonds for negatively charged residues are with the backbone atoms (O or N), while the positively charged residues hydrogen bond to suramin using the sidechain atoms. Furthermore, the non-polar residues (Gly, Ala and Pro) also have (possible) hydrogen bonds to the backbone atoms only. This corroborates the fact that the suramin binds to positively charged parts of the protein ^56^. Each of these binding sites provides a motif to DOCLASP when suramin is to be docked to a target protein.

**Table 8.** Suramin binding residues in nine non-homologous proteins from the PDB database: Interactions sorted based on the distance. R/A/LA/D: Residue number/Atom of the residue/Atom of ligand/distance between the interacting atoms (in Å). For example, ‘LYS53/NZ/O80/2.6’ means that the atom NZ from Lys53 is at 2.6 Å from the O80 of the suramin drug in PDBid:1Y4LB. The non-specific binding of suramin to phospholipase A2-like proteins (PLA2) is demonstrated by different binding sites for the homologous PLA2-X and PLA2-Z. Also, suramin binds to different sites even within the homologous subunits of PLA2-Y (marked with asterisks).

PDBid	R/A/LA/D	R/A/LA/D	R/A/LA/D	R/A/LA/D
1Y4LB,PLA2-X 3BJWA,PLA2-Y* 3BJWB,PLA2-Y* 4YV5A,PLA2-Z	LYS53/NZ/O80/2.6 PHE124/CD2/O4/2.6 SER21/OG/O25/2.7 ASN114/ND2/O35/2.7	LYS69/NZ/O54/2.8 TRP125/O/O29/3.0 SER17/OG/N19/2.8 LYS116/NZ/O35/2.7	TYR52/CD2/C60/2.8 LYS127/N/O30/3.4 LYS115/NZ/O36/2.8 TYR121/CD2/N44/3.1	ARG34/CD/C70/2.9 PRO36/CD/O28/3.5 LYS16/NZ/O34/2.8 LYS115/C/O23/3.3
1Y8EA 2H9TH 2NYRB 3GANA 3PP7B 3UR0B 4J4VA 4X3UB	ARG244/NH1/O23/2.7 GLU247/N/O77/2.5 TYR255/OH/O45/2.6 PRO152/O/N44/2.8 LYS335/NZ/O84/3.1 ARG413/NE/O84/2.7 THR63/O/N53/2.7 SER40/O/N63/2.6	LYS241/NZ/O24/2.7 ARG93/NE/O35/3.0 TYR102/OH/O30/2.8 ASP151/O/N41/3.0 ASN51/ND2/O81/3.1 PRO39/O/O79/2.8 ASN66/N/O78/2.9 HIS47/NE2/O23/2.8	LYS214/NZ/O84/2.7 ARG101/NE/O36/3.0 ARG105/NH2/O29/2.9 TYR48/OH/N53/3.0 THR26/OG1/O80/3.1 TRP42/NE1/O84/3.3 LYS67/NZ/O77/3.1 ARG20/NH1/O86/2.8	LYS220/NZ/O78/2.7 ASP243/O/N44/3.3 ARG71/NE/O23/3.0 GLY79/O/O54/3.8 GLY331/N/O82/3.3 ALA41/N/O79/3.3 GLU94/O/O85/3.4 TRP42/NE1/O81/3.0

**Figure 4.**
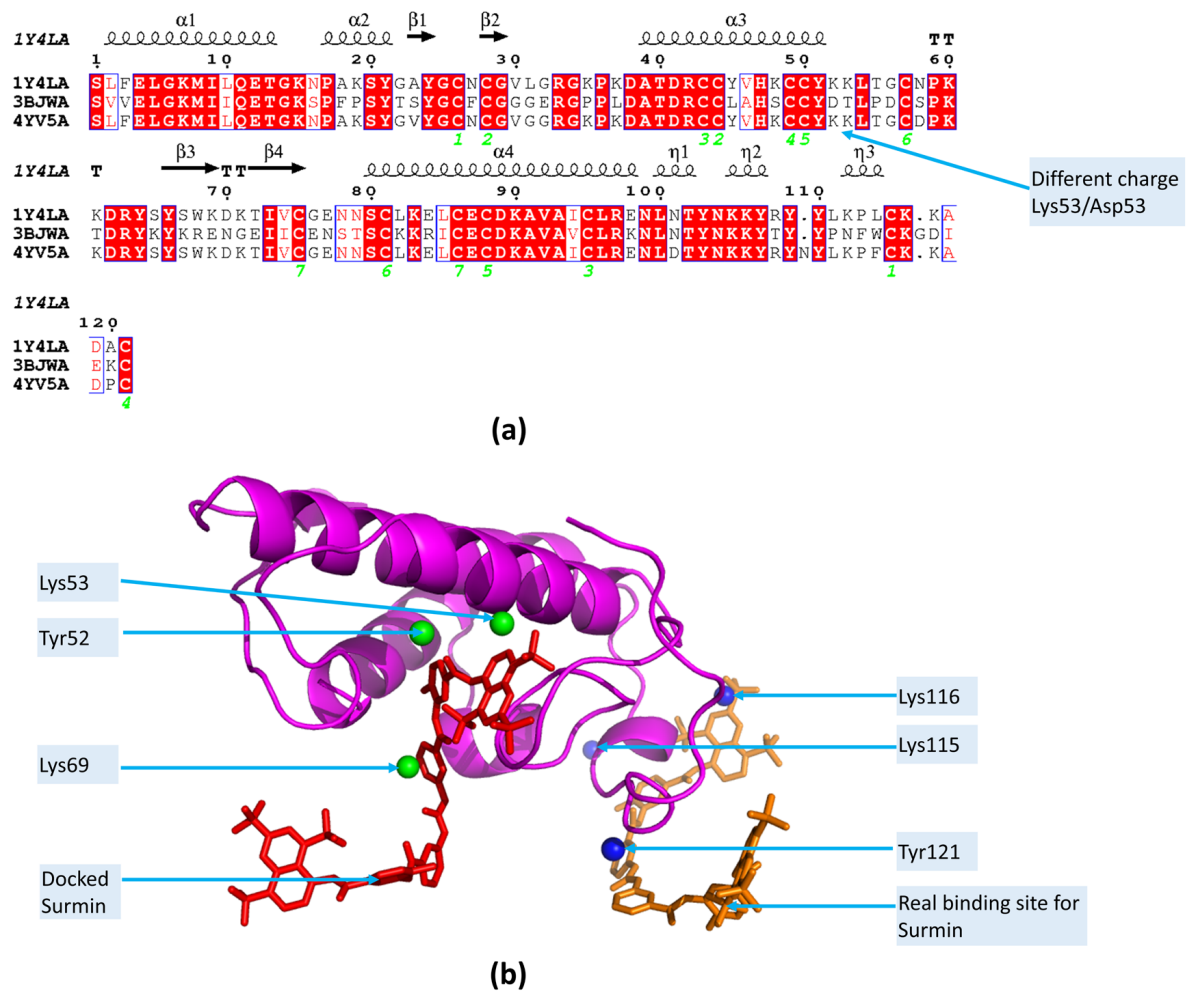
Non-specific binding of suramin to phospholipase A2-like proteins (PLA2) ( **a**) MSA of three PLA2 from poisonous vipers: (i) PLA2-X, PDBid:1Y4L from *Bothrops asper*, (ii) PLA2-Y, PDBid:3BJW from *Echis carinatus* and (iii) PLA2-Z, PDBid:4YV5 from *Bothrops moojeni* shows that PLA2-Y is significantly different from the other two proteins in the charge composition of the residues. For example, the positively charged Lys53 which makes contact with suramin in PLA2-X, and is conserved in PLA2-Z, is replaced by the negatively charged Asp53 in PLA2-Y.( **b**) The DOCLASP docked suramin to PLA2-Z based on the binding residues obtained from PLA2-X (Lys53, Lys69 and Tyr52) shows that there are two possible binding sites of suramin within PLA2-X/Z.

The extensive data available on suramin binding to different proteins highlights the complex nature of ligand docking. Three phospholipase A2-like proteins (PLA2) from poisonous vipers exist in the current PDB database which have suramin as a ligand: (i) PLA2-X, PDBid:1Y4L from *Bothrops asper* (pit viper), (ii) PLA2-Y, PDBid:3BJW from *Echis carinatus* (saw scaled viper) and (iii) PLA2-Z, PDBid:4YV5 from *B. moojeni* (Brazilian lancehead viper). Suramin has different binding sites in the two homologous subunits ( Table 8) of PLA2-Y, which is significantly different from PLA2-X and PLA2-Z ( Figure 4a). Interestingly, inspite of conserved residues in PLA2-X and PLA2-Z, suramin binds to different sites in these proteins ( Table 8). DOCLASP docking of suramin to PLA2-Z based on the binding residues from PLA2-X (Lys53, Lys69 and Tyr52) shows that this is reasonable binding site (see PLA2dockedsuramin.p1m in Dataset 1, Figure 4b).

**Figure 5.**
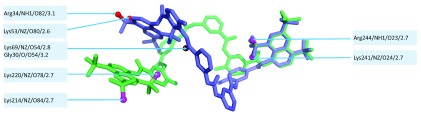
Different conformational changes in the suramin molecule on binding to different proteins Suramin molecules bound to heparin-binding site of a complement control protein from Vaccinia virus (poxvirus, PDBid:1Y8EA, in green) and the myotoxin II from Bothrops asper (venomous pit viper, PDBid:1Y4LB, in blue) shows the different conformational changes induced in the drug molecule upon binding. This significantly increases the conformational space to be sampled by computational docking methods. Different atoms of SVR are involved in the ligand binding for different proteins, although these are predominantly positively charged residues (SVR:PDBid:1Y4LB in red, SVR:PDBid:1Y8EA in mageneta).

The suramin molecule itself undergoes a significant conformational change on binding, and different proteins induce different conformational changes. Two molecules of suramin bound to different non homologous proteins (PDBid:1Y4LB and PDBid:1Y8EA - Table 1) is superimposed using DECAAF ^57^ by aligning three atoms - S17, S21 and S31 ( Figure 5). Different atoms of suramin are involved in the ligand binding for different proteins, and induce significant conformational changes in the drug. For example, the maximum distance between any two atoms in SVR:PDBid:1Y8EA is 30.211 Å (between O80 and O29), while the maximum distance in SVR:PDBid:1Y4LB is 26.4 Å (between O82 and O30). This underscores the complexity of docking algorithms, which must sample a much larger conformational space created by both the flexible binding residues and ligand ^58^.

The number of docking methods available is such that even a detailed review could only provide a partial list of currently available docking methods ^9^. The current work presents a template based, static method that leverages the spatial and electrostatic properties of the binding site. The definitive advantage of a static method can only be highlighted by emphasizing the known limitations of conformational sampling of the protein structure ^59, 60^. The problem is indeed exacerbated by the plasticity of the drug itself ^61, 62^. While DOCLASP is completely ineffectual in the absence of such a database, unlike *de novo* methods, it benefits from the burgeoning database of protein-ligand structures ^63^. The conservation of electrostatic properties, extracted using APBS/PDB2PQR, is the strongest argument in favor of DOCLASP.

There are several limitations in the method. Firstly, it can be applied to those compounds which are bound to proteins whose structures have been solved. Additionally, it is requires the structure of the apoenzyme, as this is used to extract the query motif considering the structural and electrostatic changes induced by ligand binding. However, with an ever increasing number of protein structures being solved, this is not a severe limitation since most proteins with ligands also have their apo structures solved. Furthermore, the lack of congruent matches leads DOCLASP to return a null result. This can be overcome by relaxing constraints, for example by checking for spatial congruence only. Finally, it is required to develop an energy function which will be able to discriminate poorly docked structures that have either significant steric clashes or are docked on the surface of the protein.

To summarize, this work presents an implicit method for docking ligands to proteins, in which the search and scoring are implicit in the CLASP algorithm. One significant limitation of this method is the requirement of template protein structures in complex with the given compound. As future work, I intend to incorporate the flexibility of the ligand and protein to add further discrimination.

## Data availability

The data referenced by this article are under copyright with the following copyright statement: Copyright: © 2016 Chakraborty S

Data associated with the article are available under the terms of the Creative Commons Zero "No rights reserved" data waiver (CC0 1.0 Public domain dedication). 

F1000Research: Dataset 1. Data for “DOCLASP - Docking ligands to target proteins using spatial and electrostatic congruence extracted from a known holoenzyme and applying simple geometrical transformations”, 10.5256/f1000research.5145.d125646 ^64^

## Supplementary materials

Pymol script for visualizing the docking and movie.

The file “SupplementaryPymol.p1m” contains the Pymol file for viewing vildagliptin docked to PIPLC and the file “SupplementaryMOvie.avi” contains a movie showing a 360 rotation of the ligand docked to PIPLC.

Click here to access the data.

Non-homologous set of nine proteins in the current PDB database with bound suramin:
