# Supplementary material for: DOCLASP - Docking ligands to target proteins using spatial and electrostatic congruence extracted from a known holoenzyme and applying simple geometrical transformations
Source: F1000Res. 2016 Jun 16;3:262. Originally published 2014 Oct 31. [Version 3] doi: 10.12688/f1000research.5145.3 (PMC4934513; doi:10.12688/f1000research.5145.3)
Supplement: Supplementary file 2 [file f1000research-3-9695-s0001.tgz › d056e2b0-873e-4ec6-942f-88edfab442c4.pdf]

Supplementary Table. 1: **Non-homologous set of nine proteins in the current PDB database with bound suramin:**

PDB	Length	Description
1Y4L	121	Phospholipase A2 homolog 2
1Y8E	244	Complement control protein
2NYR	271	NAD-dependent deacetylase sirtuin-5
2H9T	259	Thrombin
3GAN	157	Uncharacterized protein At3g22680
3PP7	498	Pyruvate kinase
3UR0	515	RNA-dependent RNA polymerase
4J4V	248	Nucleocapsid protein
4X3U	64	Chromobox protein homolog 7
