# Supplementary material for: DOCLASP - Docking ligands to target proteins using spatial and electrostatic congruence extracted from a known holoenzyme and applying simple geometrical transformations
Source: F1000Res. 2016 Jun 16;3:262. Originally published 2014 Oct 31. [Version 3] doi: 10.12688/f1000research.5145.3 (PMC4934513; doi:10.12688/f1000research.5145.3)
Supplement: Peer review history

# Peer review: Referee response for version 3

**DOI:** 10.5256/f1000research.9695.r14401

**Published:** 2016-06-30

**Authors:** Rahul Banerjee

**Affiliations:** 1 Crystallography and Molecular Biology Division, Saha Institute of Nuclear Physics, Kolkata, India

The paper suffers from one principal weakness, all other concerns being met. The author has significantly improved the quality of the manuscript by the addition of extra data and also modifying parts of the manuscript. As was pointed, the author needs to provide some data with regard to the precision of the docking procedure. The lack of this information remains the only lacuna in the manuscript. The author could consider taking crystal structures two homologous proteins complexed with the same ligand. One of the proteins could be homology modeled using the other as a template and the ligand docked by DOCLASP. This computaionally modeled protein could then be superposed onto the crystal structure and the RMSD calculated between the ligand atoms from the in silico model and the experimentally derived crystal structure.

I have read this submission. I believe that I have an appropriate level of expertise to confirm that it is of an acceptable scientific standard.

# Response

**Published:** 2016-06-30

**Authors:** Sandeep Chakraborty

**Affiliations:** Tata Institute of Fundamental Research, India

Dear Dr Banerjee,

    I greatly appreciate your comments.

 In my opinion, the testcase you have outlined will be more of a test for the modelling software. Let us assume the two homologous proteins are A and B, and B' is modelled using A.

 Since, DOCLASP will use the cognate binding residues in A and B (which will the same), the RMSD will be a function of how close B' is to B.

 However, I will perform this (since this is relatively easy) in a revised version in the near future.

 Thanks again,

 best,

 Sandeep

# Peer review: Referee response for version 3

**DOI:** 10.5256/f1000research.9695.r14587

**Published:** 2016-06-24

**Authors:** Amarda Shehu

**Affiliations:** 1 Department of Computer Science, George Mason University, Fairfax, VA, USA

I will be keen to see the progress of this work, particularly with regards to an integrative setting that considers various structural models and further interface with energetic minimization protocols. I do sympathize with the comment on an independent researcher aiming to share research progress in spite of large-scale evaluation settings. F1000 is the right venue for these types of dissemination.

I have read this submission. I believe that I have an appropriate level of expertise to confirm that it is of an acceptable scientific standard.

# Response

**Published:** 2016-06-26

**Authors:** Sandeep Chakraborty

**Affiliations:** Tata Institute of Fundamental Research, India

Dear Dr Shehu,

    I greatly appreciate your comments. I will keep you in the loop as I make

 changes to incorporate some sort of energy minimization into DOCLASP, hopefully

 by the end of the year.

 best wishes,

 Sandeep

# Peer review: Referee response for version 2

**DOI:** 10.5256/f1000research.6095.r13760

**Published:** 2016-05-24

**Authors:** Amarda Shehu

**Affiliations:** 1 Department of Computer Science, George Mason University, Fairfax, VA, USA

This article serves as a proof of concept. It tests the idea, previously published, that if spatial and electrostatic similarities between cognate pairs of residues are predictive of catalytic function, then aligning two proteins to match 4 cognate pairs of residues ought to identify the binding site in a novel/target protein. The alignment process additionally provides the pose of the ligand in the target protein, if the matching is conducted with a holoenzyme.

 As a proof of concept, the article is scientifically sound.

 Major observations:

1. It would be desirable to provide more testing sets. That is, expand the evaluation to more than binding of vildagliptin to PI-PLC. It is trivial to do so. The authors can use known protein-ligand complex cases, treat them as unknown effectively, and test whether they are able to reproduce the binding pose for the ligand.
2. Expanding the test set will also improve the evaluation of the proposed DOCLASP protocol. The selected case study is presented in detail, but how would one determine the effectiveness of the method at a large scale? What would be summary results on application of DOCLASP on a set of test cases?
3. The author states that a limitation of the method, as a template-based one, is the availability of protein-ligand pairs with known structures. Given the growth in structural data, this is bound to be less of a limitation now as opposed to ten years ago. However, what needs to be discussed is the sensitivity of the method. Is the author reliant on structures with good resolution? What would this be? If an X-ray structure has 2.5A or lower resolution as opposed to 1.5, should the results be trusted less?
4. Following on above, if two different poses are obtained by matching with two different holoenzymes, and one of the holoenzymes has resolution 2.5 but the other has resolution 1.5, should this be considered in what pose is recommended for the ligand in the binding protein? What is the reliance of predictions on quality of input?
5. In case of pose discrepancies from diversity of quality of holoenzymes, would energetic minimization help converge to the right pose? This would be an interesting experiment to conduct. In particular, the experiment can suggest that a specific threshold in quality is needed for the minimization to converge to the right pose.

Minor:

1. Consider using a different symbol to denote the number of holoenzymes, M, from the symbol used to denote motif, M. In particular, this can cause confusions regarding equation (5). P1...PM relates to the set of holoenzymes, whereas M1^{PM} relates to best-scoring motif extracted from the last holo-enzyme, PM.
2. Given that there are M holoenzymes, it is unclear whether the method produces M possible poses for the ligand, or if these poses are filtered or joined in some way to provide a super pose.
3. Consider using passive form rather than active "I" form when summarizing future work.

I have read this submission. I believe that I have an appropriate level of expertise to confirm that it is of an acceptable scientific standard, however I have significant reservations, as outlined above.

# Response

**Published:** 2016-06-06

**Authors:** Sandeep Chakraborty

**Affiliations:** Tata Institute of Fundamental Research, India

Dear Dr Shehu,

 I would like to thank you for taking the time to review this paper, and providing constructive criticism on the overall manuscript. I have revised the manuscript keeping these in mind. Below I outline a point-by-point response to your comments.

**This article serves as a proof of concept. It tests the idea, previously published, that if spatial and electrostatic similarities between cognate pairs of residues are predictive of catalytic function, then aligning two proteins to match 4 cognate pairs of residues ought to identify the binding site in a novel/target protein. The alignment process additionally provides the pose of the ligand in the target protein, if the matching is conducted with a holoenzyme. As a proof of concept, the article is scientifically sound.**

 I greatly appreciate the positive comment.

**Major observations: 1. It would be desirable to provide more testing sets. That is, expand the evaluation to more than binding of vildagliptin to PI-PLC. It is trivial to do so. The authors can use known protein-ligand complex cases, treat them as unknown effectively, and test whether they are able to reproduce the binding pose for the ligand.**

 Since, DOCLASP uses templates from holoenzymes, re-liganding them back would be trivially correct all the time. However, I have added two testcases. The first docks phenylthiourea to polyphenol oxidases (PPO) from walnut (JrPPO1). In a recent paper on the walnut genome sequence (http://onlinelibrary.wiley.com/doi/10.1111/tpj.13207/), DOCLASP was used to dock phenylthiourea to the modelled structure of JrPPO1. Incidentally, the structure of this protein was solved while the walnut genome manuscript was in review (PDBid:5CE9). In the current work, docking phenylthiourea to this structure resulted in almost the same pose. Secondly, a comprehensive analysis of the binding of suramin to different non-homologous proteins from the PDB database has been presented, and DOCLASP was used to dock suramin to a phospholipase A2-like protein. This highlights the non-specific binding of some ligands, and also the complexity of modelling a flexible ligand like suramin.

 2 **. Expanding the test set will also improve the evaluation of the proposed DOCLASP protocol. The selected case study is presented in detail, but how would one determine the effectiveness of the method at a large scale? What would be summary results on application of DOCLASP on a set of test cases?**

 A large scale analysis is desirable, but difficult for an individual independent researcher. Fortunately, the f1000 allows theoretically infinite revisions. This will be work in progress.

**3. The author states that a limitation of the method, as a template-based one, is the availability of protein-ligand pairs with known structures. Given the growth in structural data, this is bound to be less of a limitation now as opposed to ten years ago. However, what needs to be discussed is the sensitivity of the method. Is the author reliant on structures with good resolution? What would this be? If an X-ray structure has 2.5A or lower resolution as opposed to 1.5, should the results be trusted less? 4. Following on above, if two different poses are obtained by matching with two different holoenzymes, and one of the holoenzymes has resolution 2.5 but the other has resolution 1.5, should this be considered in what pose is recommended for the ligand in the binding protein? What is the reliance of predictions on quality of input?**

 Empirically, I would assume that the sensitivity of this method would improve with lower resolution, but I do not know how to establish that scientifically. DOCLASP also depends on APBS for computing electrostatic potential, and that is known to improve with lower resolution.

**5. In case of pose discrepancies from diversity of quality of holoenzymes, would energetic minimization help converge to the right pose? This would be an interesting experiment to conduct. In particular, the experiment can suggest that a specific threshold in quality is needed for the minimization to converge to the right pose.**

 Energetic minimization would certainly help in resolving steric clashes. Since there are

 several well established methods that can be applied to the output PDB from DOCLASP, I have not discussed these in the current paper.

**Minor: 1. Consider using a different symbol to denote the number of holoenzymes, M, from the symbol used to denote motif, M. In particular, this can cause confusions regarding equation (5). P1...PM relates to22**

**the set of holoenzymes, whereas M1PM relates to best-scoring motif extracted from the last holo-enzyme, PM.**

 Done (number of holoenzymes is denoted by Z).

**2. Given that there are M holoenzymes, it is unclear whether the method produces M possible poses for the ligand, or if these poses are filtered or joined in some way to provide a super pose.**

 If there are multiple holoenzymes, DOCLASP will generate a pose for each (assuming there is a significant match of the motif from the holoenzyme). Currently, there is no method to create a single ’super pose’ from these.

**3. Consider using passive form rather than active ”I” form when summarizing future work.**

 Done.

 I appreciate your consideration of the revised manuscript, and look forward to hearing back from you.

 best wishes,

 Sandeep

# Response

**Published:** 2016-06-20

**Authors:** Amarda Shehu

**Affiliations:** George Mason University, USA

I will be keen to see the progress of this work, particularly with regards to an integrative setting that considers various structural models and further interface with energetic minimization protocols. I do sympathize with the comment on an independent researcher aiming to share research progress in spite of large-scale evaluation settings. F1000 is the right venue for these types of dissemination.

# Peer review: Referee response for version 2

**DOI:** 10.5256/f1000research.6095.r7194

**Published:** 2015-01-20

**Authors:** Rahul Banerjee

**Affiliations:** 1 Crystallography and Molecular Biology Division, Saha Institute of Nuclear Physics, Kolkata, India

The author has presented a novel docking algorithm based on the principle that spatial and electrostatic properties in cognate pairs of residues are conserved in the catalytic site of proteins endowed with the same function. Thus, in case the crystal structure of a protein-ligand complex is available, it can be used to dock the same ligand into the active site of a homologous protein (with identical catalytic activity). The author has tested the algorithm by docking vildagliptin into the active site of PI-PLC.

1. Examination of Figure 2 and Table 5 show quite a few short contacts between the docked ligand and amino acid chains. Under such circumstances the author could consider the inclusion of an energy minimization protocol to relieve the short contacts.
2. The author considers the method validated by the above mentioned docking exercise. However, the author could consider a case where the actual crystal structure of the solution exists and the rmsd between the experimental solution and the solution obtained by DOCLASP could be provided to give some  estimate of the accuracy of the docked poses.
  Given the fact that the DOCLASP solution abounds in short contacts, further improvements could be made in the pose.
3. On page 3, two lines below Equation 4, a more explicit account of CScore could be given in the manuscript. 

I have read this submission. I believe that I have an appropriate level of expertise to confirm that it is of an acceptable scientific standard, however I have significant reservations, as outlined above.

# Response

**Published:** 2016-06-06

**Authors:** Sandeep Chakraborty

**Affiliations:** Tata Institute of Fundamental Research, India

Dear Dr Banerjee,

 I would like to thank you for taking the time to review this paper, and for your positive comments. I also apologize for the time taken to respond to your comments, a delay that took place due to the time taken to find another reviewer. Please find my detailed responses to your comments below.

**The author has presented a novel docking algorithm based on the principle that spatial and electrostatic properties in cognate pairs of residues are conserved in the catalytic site of proteins endowed with the same function. Thus, in case the crystal structure of a protein-ligand complex is available, it can be used to dock the same ligand into the active site of a homologous protein (with identical catalytic activity). The author has tested the algorithm by docking vildagliptin into the active site of PI-PLC. 1. Examination of Figure 2 and Table 5 show quite a few short contacts between the docked ligand and amino acid chains. Under such circumstances the author could consider the inclusion of an energy minimization protocol to relieve the short contacts. 2. The author considers the method validated by the above mentioned docking exercise. However, the author could consider a case where the actual crystal structure of the solution exists and the RMSD between the experimental solution and the solution obtained by DOCLASP could be provided to give some estimate of the accuracy of the docked poses. Given the fact that the DOCLASP solution abounds in short contacts, further improvements could be made in the pose.**

 Since, DOCLASP uses templates from holoenzymes, re-liganding them back would be trivially correct all the time. Energy minimizations would certainly help in resolving the steric constraints. Such methods are standard, and can be applied to the output PDB of DOCLASP, but have not been done in the current version. I will consider including this in the future. Instead, I have included two additional test cases, DOCLASP was previously applied to the binding of phenylthiourea to polyphenol oxidases (PPO) from walnut (JrPPO1) (http://onlinelibrary.wiley.com/doi/10.1111/tpj.13207/). Incidentally, the structure of this protein was solved while the walnut genome manuscript was in review (PDBid:5CE9). Here, phenylthiourea was docked to this solved structure, resulting in almost the same pose as the one in which DOCLASP docked phenylthiourea to the SWISSMODEL-modelled structure of JrPPO1. Secondly, a comprehensive analysis of the binding of suramin to different non-homologous proteins from the PDB database has been presented, and DOCLASP was used to dock suramin to a phospholipase A2-like protein. This highlights the non-specific binding of some ligands, and also the complexity of modelling a flexible ligand like suramin.

**3. On page 3, two lines below Equation 4, a more explicit account of CScore could be given in the manuscript.**

 I have modified the methods section to give a more explicit explanation of CScore.

 I appreciate your consideration of the revised manuscript, and look forward to hearing back from you.

 best wishes,

 Sandeep
